# Receptor-Dependent and Independent Regulation of Voltage-Gated Ca^2+^ Channels and Ca^2+^-Permeable Channels by Endocannabinoids in the Brain

**DOI:** 10.3390/ijms22158168

**Published:** 2021-07-29

**Authors:** Tomasz Boczek, Ludmila Zylinska

**Affiliations:** Department of Molecular Neurochemistry, Faculty of Health Sciences, Medical University of Lodz, 92215 Lodz, Poland; tomasz.boczek@umed.lodz.pl

**Keywords:** endocannabinoids, calcium, calcium channels, cannabinoid receptors, signal transduction, synaptic transmission

## Abstract

The activity of specific populations of neurons in different brain areas makes decisions regarding proper synaptic transmission, the ability to make adaptations in response to different external signals, as well as the triggering of specific regulatory pathways to sustain neural function. The endocannabinoid system (ECS) appears to be a very important, highly expressed, and active system of control in the central nervous system (CNS). Functionally, it allows the cells to respond quickly to processes that occur during synaptic transmission, but can also induce long-term changes. The endocannabinoids (eCBs) belong to a large family of bioactive lipid mediators that includes amides, esters, and ethers of long-chain polyunsaturated fatty acids. They are produced “on demand” from the precursors located in the membranes, exhibit a short half-life, and play a key role as retrograde messengers. eCBs act mainly through two receptors, CB1R and CB2R, which belong to the G-protein coupled receptor superfamily (GPCRs), but can also exert their action via multiple non-receptor pathways. The action of eCBs depends on Ca^2+^, but eCBs can also regulate downstream Ca^2+^ signaling. In this short review, we focus on the regulation of neuronal calcium channels by the most effective members of eCBs-2-arachidonoylglycerol (2-AG), anandamide (AEA) and originating from AEA-N-arachidonoylglycine (NAGly), to better understand the contribution of ECS to brain function under physiological conditions.

## 1. Introduction

Ca^2+^ is a universal signaling molecule acting at several molecular levels to control a wide variety of cellular processes, including regulation of gene transcription, cell proliferation and development, as well as mediating short- and long-term adaptive responses. In neurons, the diversity of the responses to Ca^2+^ signal depends on the ability to activate or suppress specific intracellular signal transduction pathways. Given the complexity of the nervous system, the intensity and duration of Ca^2+^ transients usually depend on the coordinated influx through voltage-gated calcium channels (VGCCs) and calcium-permeable-channels, both located in the plasma membrane, the release from internal stores and the efficiency of the re-uptake mechanisms to restore resting membrane potential [1,2]. To date, the contribution of numerous regulators of Ca^2+^ signaling located in the plasma membrane and intracellular organelles has been documented, and various chemical compounds targeting ion channels, G-protein coupled receptors, pumps and enzymes have been identified.

Among others, the endogenous cannabinoid system (ECS) in the brain and its roles in physiology, behavior and potential pathological involvements have been extensively investigated and demonstrated in the regulation of CNS function [3,4,5]. The significance of cannabinoids in neurosignaling is indicated by the presence of endogenous cannabinoids (eCBs) and cannabinoid receptors in different brain cells, i.e., neuron, astrocytes, and microglial cells [6,7,8]. The ECS can act as a negative feedback mechanism to suppress synaptic transmission; therefore, it may regulate physiological processes such as motor control, cognition, emotion, behavior, learning and memory, or mood [9,10]. The endocannabinoids belong to a family of bioactive lipid mediators, which includes amides, esters, and ethers of long-chain polyunsaturated fatty acids. They are produced from the precursors located in the membranes and released mainly by the diffusion, playing a key role as retrograde messengers. The most effective appear to be derivatives of arachidonic acid: 2-arachidonoylglycerol (2-AG), anandamide (AEA) and originating from AEA N-arachidonoylglycine (NAGly) [6,11]. The crucial feature in the action of eCBs is the rate of their metabolism and generally they have a very short half-life. It has been well documented that metabolism of eCBs depends on Ca^2+^, but eCBs can also regulate downstream Ca^2+^ signaling. Physiologically, the ECS exhibits a protective role by inhibiting neurotransmitter release and decreasing Ca^2+^ entry into the cells via calcium channels [12]. Endocannabinoids act mainly via two receptors, CB1R and CB2R, which are the members of class A of the G-protein coupled receptor family (GPCRs) [13]. Based on the mechanism of action, G protein-coupled receptor 55 (GPR55) has recently been added to the eCBs receptor family, although there is no significant sequence similarity between GPR55 and CB1R or CB2R2 [14].

A growing body of evidence indicates that eCBs can also modulate signal transduction pathways activating the “non-canonical” cannabinoid receptors, i.e., other than CB1R and CB2R [15]. In this short review, we focus on the regulation of calcium channels by eCBs mediated by both receptor and non-receptor signaling pathways, which is relevant to understanding the contribution of ECS to brain function under physiological conditions.

## 2. Endocannabinoid System in the Brain

The ECS consists of endogenous ligands produced locally from the membrane lipids, enzymes involved in their synthesis and degradation, and two main receptors—CB1 and CB2 [5,16]. However, a growing amount of evidence suggests that several GPR proteins (GPR18, GPR55, GPR119) can also exhibit canonical receptor-like activity [17,18,19,20]. Besides classical receptors, endocannabinoids have been described to interact, although with lower specificity, with a number of targets including other membrane receptors (opioid, glycine, metabotropic glutamate receptors) and ion channels [21,22]. Endocannabinoids are synthesized locally on demand within the plasma membrane and can act in autocrine and/or paracrine fashion. eCBs are produced in postsynaptic membranes in response to increased intracellular Ca^2+^ concentration, traverse to the presynaptic terminal and activate CB receptors [23,24]. Retrograde eCBs signaling is mostly inhibitory due to a reduction in neurotransmitter release [25].

### 2.1. Metabolisms of Endocannabinoids

**Figure 1:**
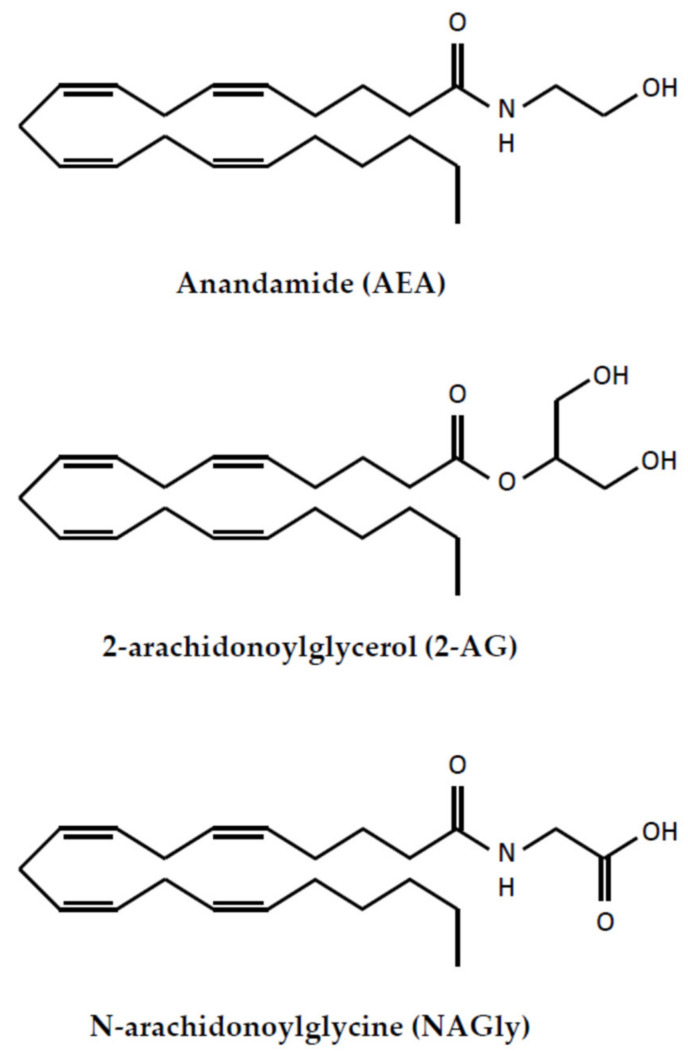
The structure of endocannabinoids.

In general terms, endocannabinoids are a very large group of phospholipid-derived compounds, but their biological activity differs significantly. It should be noted that eCB biosynthesis is controlled by calcium and cAMP [26]. The eCBs can be synthesized from arachidonic acid (AA), omega-6 and omega-3 polyunsaturated fatty acids (PUFAs) [27,28,29,30]. eCBs are short-lived signaling molecules and their lifespan is limited by the uptake into neural cells or by enzymatic hydrolysis [31]. The most prominent and well-characterized are 2-AG, AEA and its derivative—NAGly (Figure 1).

2-AG can be synthesized postsynaptically in several ways depending on the availability of a particular substrate and the enzyme. The most prominent substrate is diacylglycerol (DAG) that can be hydrolyzed by two diacylglycerol lipases: DAGLα and DAGLβ, but 2-AG can also be produced from omega-3 and omega-6 polyunsaturated fatty acid (PUFA) or arachidonic acid (AA) [8,28,32]. 2-AG is degraded by the presynaptically located monoacylglycerol lipase (MAGL), which is a main enzyme controlling duration of 2-AG-mediated signaling, but also by fatty acid amide hydrolase (FAAH) or postsynaptic α,β-hydrolase 6 and 12 [3,33]. Additionally, 2-AG is the substrate for cyclooxygenase 2 (COX-2), lipoxygenases (LOX), and cytochrome P450 epoxygenases (CYP450) [34,35].

Similar to 2-AG, anandamide is formed by multiple pathways by the cleavage of a phospholipid precursor. However, the AEA level in the brain has been estimated to be even three times lower than 2-AG [36]. Nonetheless, the overall distribution of AEA and 2-AG in different brain areas appears to be similar; the highest in brainstem, striatum and hippocampus and lower in cortex, diencephalon and cerebellum [37]. One of the pathways for AEA synthesis is a two-step process involving Ca^2+^-dependent N-acyltransferase and phospholipase D (NAPE-PLD) [38]. Another possibility has been revealed in the mouse nervous system where AEA synthesis involved sequential actions of two enzymes: α/β-hydrolase 4 (Abh4) and glycerophosphodiesterase GDE1 [39]. AEA is produced postsynaptically but, in this case, it is a downstream product of phospholipase A2 or PLC [40]. The major AEA degrading enzyme is fatty acid amide hydrolases 1 and 2 (FAAH1, 2) [41]. AEA is also metabolized by eicosanoid synthesizing enzymes LOX and COX-2 [34,35].

N-arachidonoyl glycine (NAGly) is another endogenous cannabinoid that can be formed by two pathways; a conjugation of arachidonic acid and glycine or oxygenation of AEA via the sequential enzymatic reaction of alcohol dehydrogenase (ADH) and aldehyde dehydrogenase [29,42]. Interestingly, NaGly has been shown to act as an endogenous inhibitor of fatty acid amide hydrolase (FAAH), thereby increasing the concentration of AEA [43]. NAGly can be metabolized by COX-2 to prostaglandin H_2_-glycine (PGH_2_-Gly) or by FAAH to arachidonic acid and glycine [44,45].

### 2.2. Classical and Non-Classical GPCRs for eCBs

**Figure 2:**
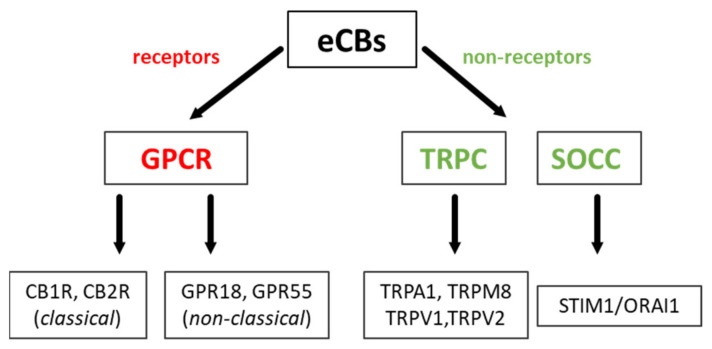
The schematic presentation of endocannabinoid action. GPCR—G protein-coupled receptors, TRPC—transient receptor potential channel, SOCC—store-operated calcium channel.

The schematic receptor and non-receptor action of endocannabinoids is presented in Figure 2. The most widely characterized are two principal types of classical cannabinoid receptors—CB1R and CB2R, both ubiquitously present in all major brain cell types: neurons, astrocytes, oligodendrocytes, and microglia. They act mainly by G_i_ and G_0_ classes of G proteins, which suppress adenylate cyclase (AC) activity, reduce cAMP production, but can also modulate potassium and calcium channels. Moreover, there is an increasing amount of data showing several alternative regulatory pathways (see below). In the brain, these two receptors differ in localization and affinity for particular eCBs.

**Figure 3:**
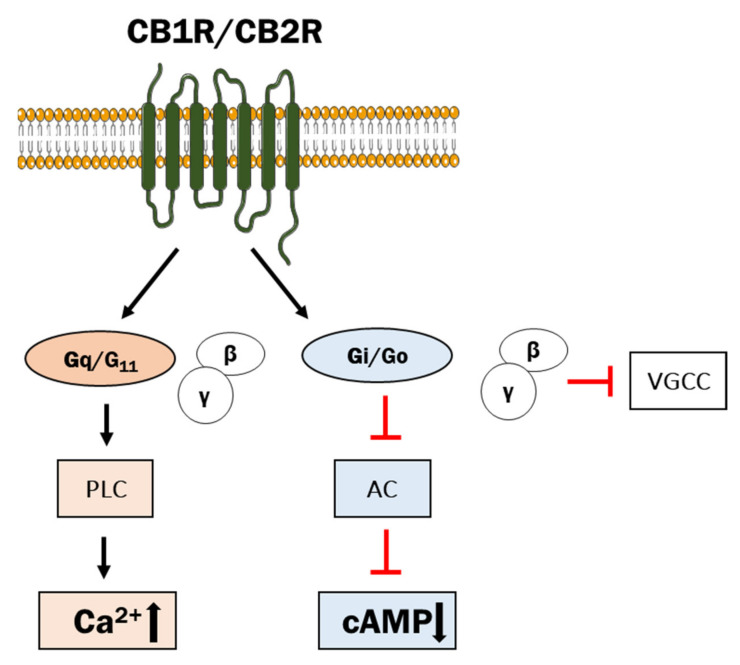
The scheme of receptor action of endocannabinoids. PLC—phospholipase C, AC—adenylyl cyclase. Inhibitory action is indicated by the red arrow.

The CB1R, encoded by the *CNR1* gene, is the most prevalent and is located in the cortex, hippocampus, cerebellum, thalamus, amygdala, basal ganglia, striatum, globus pallidus, substantia nigra and olfactory areas, and represents the GPCR with the highest expression in the CNS [3,4,46]. For that reason, it may be recognized as the principal regulator of neuronal function with diverse effects on neuronal responses. The CB1R is thought to play a key role in long-term synaptic depression (LTD) that involves a long-lasting decrease in neurotransmitter release [47]. The CB1R has been detected in axons and nerve terminals, but also in neuronal cell bodies [48,49,50]. The CB1R is the most abundantly expressed GPCR in the CNS and can be considered as the principal regulator of neuronal function with diverse effects on neuronal responses [51]. There are also reports describing the presence of CB1R polymorphic forms, but analysis of association between gene variants and brain structure and function has so far yielded ambiguous results [52,53,54,55]. Functionally, the CB1R regulates dopaminergic and GABAergic neurons via second messenger systems [56,57]. The CB1R is mainly coupled to pertussis toxin-sensitive G_i/o_ proteins that regulate cAMP levels and subsequently PKA-mediated pathways, but can also couple to G_s_, G_q_, and G_12/13_ depending on the cell type [58,59] (Figure 3). Interaction of the CB1R with the G_i_ βγ-subunit reduced intracellular Ca^2+^ concentration by inhibiting N- and P/Q-type voltage-gated calcium channels [50]. Activated CB1R suppressed the neuronal release of acetylcholine, noradrenaline, dopamine, serotonin, GABA, glutamate and aspartate [17].

The CB1R-induced activation of PLC and mobilization of Ca^2+^ from internal stores involving G_q/11_ has been shown in astrocytes [60]. In astroglial cells, eCB activation of the CB1R increased intracellular Ca^2+^concentration, likely via G_q_ protein coupling rather than G_i/o_ [61]. Downstream signaling of the CB1R is associated with the activation of several protein kinases, including extracellular signal-regulated kinases (ERK), focal adhesion kinases (FAK), c-Jun N-terminal kinase 1/2, and the mitogen-activated protein kinases (MAPK) p42/p44 and p38 [62].

CB2R, encoded by the *CNR2* gene, is expressed in microglia and astrocytes, and postsynaptically in some populations of neurons. However, in physiological conditions CB2R mRNA is about 100–300 times lower than CB1R mRNA [63,64,65,66]. CB2R was found in the brainstem, microglia and astrocytes [67,68]. Brain CB2R is inducible and is up-regulated in response to various insults [69,70]. Similar to the CB1R, CB2R can activate the same downstream signaling pathways, including MAP kinases and phosphatidyl- inositol-3-kinase (PI3K)/Akt, and ultimately suppressing neuronal activity [71,72]. The CB2R exerts its effects partially through stimulation of PLC/DAG/IP_3_ signaling pathways with a subsequent increase in intracellular calcium concentration [73].

The complexity of CB receptor-mediated signaling is additionally potentiated by the ability to form homo- and heterodimers. In case of the CB1R, heterodimers can be formed with CB2R, but also with other receptors for adenosine, dopamine, serotonin and opioid receptors (reviewed in [65,74]). It should be noted that heterodimerization can lead to alterations in signaling pathways. For example, dimeric form of CB1R/D2 can switch the signaling of the CB1R and dopamine receptor D2 from G_i/o_ to G_s_, thereby generating the opposing effect, i.e., an increase in cAMP levels [75].

Results obtained in last years have shown that besides classical receptors, eCBs can act on several orphan GPCRs in the brain. The most effective are GPR18 and GPR55, but some of endocannabinoids may activate or block one type of GPRs more potently than the other one.

GPR18 belongs to class A of the G-protein-coupled receptors; however, its structural and phylogenic background is different from CB_1_ and CB_2_ [76]. The high expression of GPR18 was detected in hypothalamus, cerebellum and brain stem, while lower was in cortex, thalamus and striatum [77]. The mRNA for GPR18 was identified in microglia, astrocytes, primary neurons, and some neuron-like cell lines, and the presence of the receptor at a protein level was confirmed in primary astrocytes, hippocampal neurons and microglia [78]. Additionally, GPR18 and CB2 have been recently shown to be co-expressed and form heterodimers in microglia [79]. Findings from many studies indicate that GPR18-dependent signaling is complex and sometimes controversial, as the effect of GPR18 activation by particular ligands strongly depends on the investigated biological endpoint. Depending on whether GPR18 is activated by AEA or NAGly, downstream signaling may lead to decrease in cAMP concentration, reduction in ERK phosphorylation, increase in intracellular Ca^2+^concentration and MAPK activity, showing the potential participation of Gα_i/o_ and Gα_q_ protein [80,81].

GPR55 is a seven transmembrane G protein-coupled receptor and it is now identified as a putative “type 3” cannabinoid receptor [20,82]. In the CNS, GPR55 is found in several regions of the brain including the caudate nucleus, putamen, hippocampus, thalamus, pons, cerebellum, frontal cortex and hypothalamus [76,83]. GPR55 stimulation by lysophosphatidylinositol (LPI) activates G_12/13_ and G_q/11_ proteins, with subsequent activation of ERK and increase in intracellular Ca^2+^ concentration suggesting that LPI is a natural ligand for GPR55 [84,85]. It has been shown that GPR55 is also a target for AEA, which can trigger the signaling pathways with a potency similar to the one activating CB1R and CB2R [19]. Up to now, the G_q_, G_12_ and G_13_have been identified to transmit signal from GPR55 [21]. Another endocannabinoid, NAGly, was found to be an agonist for GPR55, which activated MAP kinases and produced Ca^2+^mobilization from the endoplasmic reticulum by inositol-3-phosphate receptors [22].

## 3. Regulation of Voltage-Gated Ca^2+^ Channels and Other Ca^2+^-Permeable Channels by eCBs

One of the most important roles of eCBs in the CNS is a regulation of synaptic plasticity, which engages a large number of biological targets such as receptors, channels, neurotransmitters and enzymes [4,86]. Release of neurotransmitters at the excitatory and inhibitory synapses can lead to transient processes, known as depolarization-induced suppression of inhibition (DSI) and depolarization-induced suppression of excitation (DSE), as well as can induce the persistent effects known as long-term potentiation (LTP) and long-term depression (LTD) [25,87]. These events involve calcium and calcium channels that are key modulators of synaptic plasticity which, in parallel, can be controlled by eCBs [88].

Physiologically, intracellular Ca^2+^ concentration is regulated by balancing calcium influx through the plasma membrane with its active efflux and sequestration into internal stores. Calcium channels responsible for the increase in intracellular free Ca^2+^ comprise voltage-gated calcium channels (VGCC), receptor-operated channels (ROC), store-operated channels (SOC) and transient receptor potential (TRP) channels [89]. The VGCCs are virtually distinguishable from other Ca^2+^-permeable channels by their biophysical properties and the mechanism of action [90]. They are activated by the changes in the electrical membrane potential and have a crucial role in the nerve tissue allowing a rapid and coordinated depolarization in response to voltage change. Similar to the highly selective VDCCs, TRP channels generate inward current that is almost exclusively carried by Ca^2+^. The opening of these channels leads to membrane depolarization and intracellular Ca^2+^ rise, both of which triggering neuronal response [91,92]. The mechanism of TRP channel activation is independent from membrane voltage; however, shifting the potential toward more negative may increase TRPC-sensitive Ca^2+^ influx and enhance electrochemical driving force for Ca^2+^, particularly in non-excitable cells. Voltage-independent Ca^2+^ influx through ROC and SOC is activated in response to depletion of the Ca^2+^ stores by ligand-gated calcium channels, including IP_3_ receptors and ryanodine receptors [93,94]. Since calcium channels control intracellular Ca^2+^ concentration, and Ca^2+^ concentration controls cell functions, critical for understanding of neuronal signal integration is the identification of factors regulating their activity. Among others, endocannabinoids acting through CB1 or CB2 receptors or in a receptor-independent manner have been shown to regulate several Ca^2+^ channels. The changes in the activity of Ca^2+^-permeable channels evoked by eCBs could also be functionally coupled to the activity of Ca^2+^-activated K^+^ channels.

### 3.1. VGCCs

In excitable cells, there are five main types of VGCCs, N, L, P/Q, R and T, which display different single-channel conductance [95]. VGCCs are important mediators of depolarization-evoked release of neurotransmitters. They are classified into three major channel categories: high voltage-activated (L- and P/Q-types), intermediate voltage-activated (R-type) and low voltage-activated (T-type). Based on the subunit composition, ten members of VGCCs can be now distinguished: L-type (Ca_v_1.1–Ca_v_1.4), P/Q-type (Ca_v_2.1), N-type (Ca_v_2.2), R-type (Ca_v_2.3) and T-type (Ca_v_3.1-Ca_v_3.3) [96]. The L-type participates in Ca^2+^-induced long-term potentiation (LTP) in dendritic spines and postsynaptic dendrites, while the P/Q-type located presynaptically generates inward Ca^2+^ currents, initiates neurotransmitter release and regulates presynaptic plasticity [97].

**Figure 4:**
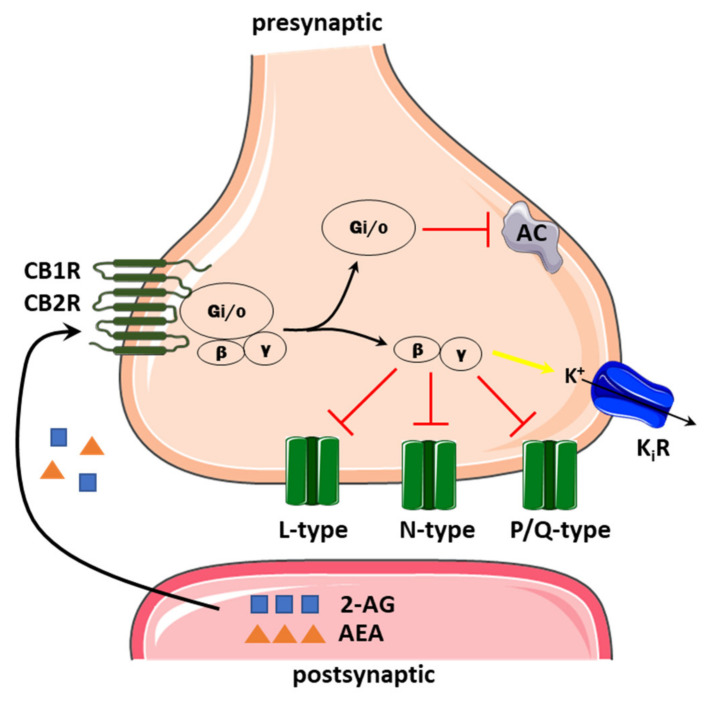
The schematic representation of endocannabinoid action on presynaptic Ca^2+^ channels. Endocannabinoids synthesized postsynaptically act on presynaptic CB1/CB2 receptors coupled to Gi/o, which may decrease cAMP level by inhibiting adenylyl cyclase (AC). The βγ inhibits L, N and P/Q-type calcium channels but activates inwardly, rectifying the potassium channel (KiR, yellow arrow). Inhibitory action is indicated by the red arrow.

A general profile of the regulation by eCBs shows an inhibitory effect on VGCCs, thereby eCBs can potentially reduce presynaptic Ca^2+^ influx and suppress the release of neurotransmitters (Figure 4) [31,98]. eCBs modulate nearly all types of neuronal VGCCs; however, there are some differences between brain regions and cell types, as well as underlying molecular mechanisms. One of the most interesting is the regulation of VGCCs by CB receptor-dependent and independent ways. Activation of the CB1R appears to be a prevalent mode of action, but different G subunits may be engaged. Although the main eCB activity involves inhibitory G protein (G_i/o_), it is now well established that G_βγ_ subunits can directly interact with and inhibit the high-voltage-activated Ca^2+^ channels regulating neurotransmitter release at most synapses [99].

L-type channels form the largest family of the VGCCs and, due to the specific expression pattern in the brain, they play a significant physiological role in diverse cellular processes including neuronal development, regulation of cell cycle, neurotransmission or gene expression. A number of studies have shown that activity of L-type of VGCCs can be regulated by eCBs, mainly by the CB1R [100,101]. Electrophysiological and pharmacological data showed that activation of dendritic L-type Ca^2+^ channels and the subsequent release of 2-AG acting on the presynaptic CB1 receptors triggered retrograde short-term depression (STD) in bed nucleus of the stria terminalis (BNST) neurons and in spiny neurons in corticostriatal slices [102]. In both rodents and humans, the BNST has prominent white matter connectivity with the amygdala via the stria terminalis, as well as with the inferior hippocampus, hypothalamus, thalamus, and infralimbic/ventromedial prefrontal cortex. BNST mediates different defensive behaviors, and can play an integrative modulatory role in fear memory formation, which is controlled by endocannabinoids [103]. L-type Ca^2+^ channels were inhibited by CB1 receptor agonist in cat brain arterial smooth muscle cells, which express mRNA and CB1 receptor protein [104]. AEA has been shown to inhibit L-type Ca^2+^ channels independently, as well as negatively regulate 2-AG biosynthesis and physiological effects in striatum, underscoring its essential role in the regulation of synaptic transmission [105]. It was also found that 2-AG and AEA controlled different forms of plasticity in the extended amygdala of rats acting via the CB1R. One of them involved activation of dendritic L-type Ca^2+^ channels and due to subsequent release of 2-AG and binding to the presynaptic CB1 receptors triggered retrograde short-term depression. Interestingly, in striatum retrograde 2-AG/CB1R-signaling mediated both short- and long-term depression [106]. The voltage-dependent L-type Ca^2+^ current (I_(Ca,L)_) was also inhibited by a structural hybrid between capsaicin and anandamide named arvanil [107]. In a mouse neuroblastoma and rat glioma hybrid cell line, NG108-15, arvanil decreased I_(Ca,L)_ in a concentration-dependent manner without interacting with either vanilloid or cannabinoid receptors. The modulation of other VGCCs namely N- and P/Q-type by eCBs was described in several cell lines, expression systems, in cultured neurons and confirmed in the brain slice preparations, as well as in animal models [108,109,110,111,112,113]. Most of the data suggest that the selective regulation of N-type could represent a general mechanism for the CB1R-triggered and G_i_/_0_ protein-mediated retrograde inhibition, but it should be noted that some neuronal processes involve cooperative action of various types of VGCCs [114]. Additionally, it may depend on the differences in the expression level of the CB receptors. For example, it was shown that CB1 activation promoted neurite outgrowth via a mechanism that requires calcium influx into neurons through N- and L-type calcium channels [115]. In the brain slice preparation AEA substantially depressed corticostriatal glutamatergic synaptic transmission onto striatal neurons [116]. Activation of presynaptic CB1 receptors via G_i/o_ protein-coupled signaling pathway inhibited N-type Ca^2+^ channel activity decreasing glutamate release. Study on transmission at the granule cell to Purkinje cell synapse showed that cannabinoids modulated N-type channels stronger than P/Q-type or R-type [111]. However, their participation in Ca^2+^ flow has been estimated to account for ∼30%, thus P/Q-type calcium channels were mainly responsible for most of the cannabinoid-mediated effects. A growing body of evidence indicates that eCBs can exhibit dual mode of action—at lower concentration (1 µM) they act through CB receptors, but at higher (>1 µM) they can directly inhibit N-type VGCCs [117,118].

Low-voltage-activated T-type Ca^2+^ channels (Ca_V_3) are important regulators of the transmission of nociceptive information in the primary afferent pathway, and are also a target for eCBs regulation [119,120]. However, inhibition of T-currents by eCBs was independent from the activation of CB1/CB2 receptors, with potencies in the high nanomolar and low micromolar range [121,122,123]. AEA was the first identified ligand showing direct action on T-channels at submicromolar concentrations [124]. The binding of AEA stabilized T-channels in the inactivated state, significantly decreasing T-currents associated with neuronal firing activities and reduced the number of channels available to open during depolarization thus limiting further calcium entry [125]. Additionally, NAGly, a derivative of AEA, can strongly inhibit Ca_V_3.1, Ca_V_3.2, and Ca_V_3.3 channels, with IC_50_ below 1.0 μM and the analgesic effect of some of these compounds depends on Cav3.2 channels [126].

### 3.2. TRP Channels

Transient receptor potential (TRP) channels are a superfamily of ubiquitously expressed cation channels that consists of 28 members and, based on sequence homology, they are grouped into six subfamilies: TRPC (Canonical), TRPV (Vanilloid), TRPM (Melastatin), TRPA (Ankyrin), TRPML (Mucolipin), and TRPP (Polycystic) [92]. They are located in the plasma membrane and in the membrane of intracellular organelles, and are activated by numerous physical and/or chemical stimuli. Cation selectivity depends on the subfamily and subtype, however for all TRP channels, the activation leads to membrane depolarization or increase in intracellular Ca^2+^ concentration. There is growing evidence indicating that several TRP subtypes referred to as “ionotropic cannabinoid receptors” may represent novel molecular targets for cannabinoids [127,128,129,130,131]. Endocannabinoids such as AEA and 2-AG have been shown to activate TRP channels with subsequent depolarization and Ca^2+^ influx [132,133]. Among potential eCB targets are TRPA1, TRPV1, TRPV2 and TRPM8 channels. TRPV2 and TRPV4 are the two main isoforms present in the blood–brain barrier (BBB). TRPV2 plays a main role in the regulation of BBB permeability, thus its agonists may exert a protective effect via preserving or rescuing BBB integrity [134]. A growing body of evidence suggests that some channels can functionally interact with each other. For example, although TRPA1 and TRPV1 can function independently, they are co-expressed and about 30% of TRPV1-expressing sensory neurons also exhibit TRPA1 expression [135]. TRPV1 has been reported to influence several features of TRPA1 channels, such as voltage–current relationship and open probability [135]. Both channels can cross-desensitize one another when acted upon by their respective agonists [136]. Moreover, co-activation of the CB receptors by eCBs and TRP channels may sometimes complicate the separation between these pathways [137].

TRPV1 is Ca^2+^ permeable channel which can act as a trigger for Ca^2+^-induced cell signaling. TRPV1 channels are highly expressed in primary sensory afferents, but are also present in the brain, contributing to many basic neuronal functions including resting membrane potential, neurotransmitter release and synaptic plasticity [138]. Moreover, they are functionally active and participate, directly or indirectly, in the long-term regulation of synaptic strength during brain development [139]. eCBs have been demonstrated to affect synaptic depression acting throughTRPV1. Hence, eCBs action through TRPV1 may be linked with the initial stages of learning and new associative memories [140]. The functional coupling between the CB1R and TRPV1 may also arise, at least in part, from their strong co-localization, as detected in sensory neurons of the spinal cord and dorsal root ganglia [141]. Endocannabinoid- and TRPV1-mediated regulation of synaptic strength at central synapses was demonstrated in the dentate gyrus and the nucleus accumbens, where TRPV1 activation by AEA induced postsynaptic LTD in a CB1R-independent manner [142,143]. There is also evidence that 2-AG contributes to PLC-dependent TRPV1 activation and TRPV1-mediated antinociceptive signaling in the brain [144,145]. There are many reports showing that the eCB system not only promotes LTD but also LTP [146,147,148,149]. In the dorsal striatum induction by a low number of paired stimulations was sufficient to increase synaptic efficacy through a signaling pathway which activated the CB1R and TRPV1 and elevated 2-AG, which prolonged application systematically yielded to LTD [148,150]. Both AEA and 2-AG have been shown to activate TRPV4 receptors, but AEA antagonized TRPM8 in dorsal root ganglion neurons at submicromolar concentrations in CBR-independent way [151,152,153].

### 3.3. Calcium-Permeable Channels

Some TRPs are expressed in the membranes of intracellular organelles, such as the endoplasmic reticulum, which can induce an additional Ca^2+^ influx via store-operated channels [154,155]. Store-operated calcium channels (SOCs) are another type of calcium channels regulating a wide variety of cell functions and exhibiting a unique mechanism of action and substrate specificity [156,157]. They are activated by a sequence of events initiated at a cell surface. Stimulation of GPCRs or tyrosine kinases receptors (TrK) results in a cleavage of phosphatidylinositol 4,5-bisphosphate (PIP_2_) by phospholipase C (PLC) and produces soluble inositol 1,4,5-trisphosphate (IP_3_) and membrane-bound diacylglycerol (DAG). Binding of IP_3_ to the specific receptors (IP_3_Rs) located in the endoplasmic reticulum (ER) leads to the release of Ca^2+^ to the cytoplasm. Depletion of the ER luminal Ca^2+^ triggers stromal interaction molecule (STIM) proteins, which are intralumenal Ca^2+^ sensors, to accumulate at the ER-plasma membrane junctions, and to open calcium channels in the plasma membrane formed by the ORAI proteins [158]. There is now strong evidence that in the process named store-operated calcium entry (SOCE), active channels are formed by a dimer of STIM and a hexamer of ORAI subunits [159]. Two isoforms, STIM1 and STIM2, and three isoforms, ORAI, ORAI2 and ORAI3, are expressed in the nervous system, but the composition of active store-operated calcium channel (SOCC) can vary between particular brain areas [160].

**Figure 5:**
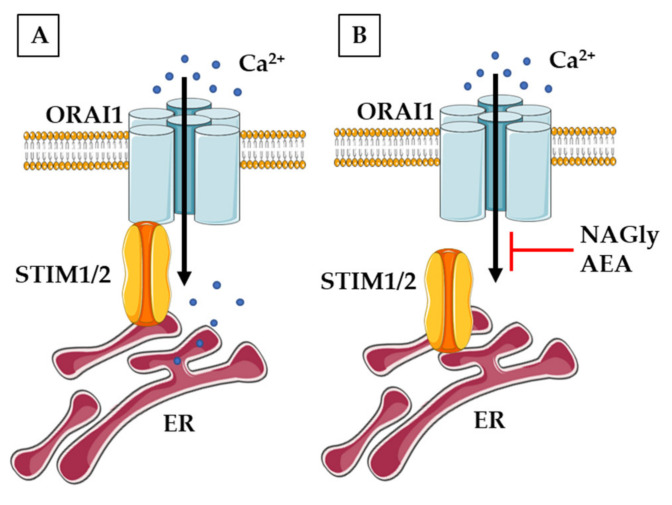
The overview of neuronal mechanism of non-receptor endocannabinoids action on SOCE. (**A**) The simplified view of SOCE in physiological conditions. (**B**) NAGly and AEA can block the interaction of STIM proteins with ORAI channel and inhibit SOCE.

Due to close functional cooperation between TRP and SOCCs channels, as well as the confirmed modulation of TRPs by eCBS, increased attention has been recently given to the potential role of endocannabinoids in the control of SOCE (Figure 5). The regulation of SOCCs by eCBs was demonstrated for the first time in non-neuronal cell lines (human umbilical vein-derived endothelial cells, pancreatic *β*-cells and rat basophil leukaemia cells) showing that NAGly, but not AEA, effectively reduced SOCE by preventing the interaction between STIM1 and Orai1 [161]. Inhibitory action of NAGly was reversible, concentration- and time-dependent. Further studies confirmed that NAGly can affect SOCE in several other models in a manner independent of cannabinoid CB1/2 receptors. Live-cell Ca^2+^ imaging showed that AEA and NAGly depressed SOCE in cultures of primary mice cortical neurons, and this effect was insensitive to PTX, indicating that G_i/o_ proteins were not involved [162]. In addition, NAGly was able to release Ca^2+^ from the ER potentiating the passive calcium leak. Similar results were obtained in another study on mice primary cortical cells confirming that NAGly can serve as an endogenous modulator interfering with the core machinery of SOCE [163].

In isolated dorsal horn neurons, NAGly has also been shown to act as a competitive antagonist at the glycine binding site of the N-methyl-D-aspartate (NMDA) receptor, thereby depressing excitatory NMDA-dependent synaptic transmission [164]. It is commonly known that Ca^2+^ influx through the NMDA receptor is required for the induction of LTP either through the release of Ca^2+^ from the internal stores or direct activation of Ca^2+^-sensitive downstream targets [165]. Binding to NMDA receptor may also indirectly depolarize neurons to increase membrane excitability. In pyramidal cells of the basolateral amygdala, NMDA receptor-mediated synaptic transmission preferentially activates VGCCs that evoke large depolarizing spikes [166]. This supports the model where the NMDA receptor-dependent neurotransmission is mediated by the activation of VGCCs.

An intriguing relationship exists between ECS and the ionotropic NMDA receptors (NMDARs). They are ligand-gated channels highly permeable to Ca^2+^. Based on the structure, three main types of subunits have been characterized: GluNR1 (with eight isoforms), GluNR2 (with four subtypes) and GluNR3 (with two subtypes) [167,168]. Functionally, the receptors form heterotetrameric complexes containing a pair of GluNR1 subunits and two GluNR2 and/or GluNR3 subtypes [169]. In the CNS, NMDARs by regulating ion flux, participate in the synaptic plasticity, learning, memory and cognition.

A growing body of evidence indicates that NMDA receptor function is under control of endocannabinoids acting mainly through the CB1R. This action can presynaptically decrease the release of glutamate or postsynaptically directly inhibit NMDA receptor current [170,171]. On the other hand, NMDARs contribute to the generation of eCBs signaling through elevating of postsynaptic Ca^2+^ concentration [172]. Although eCBs release is mainly driven by the activation of G_q/11_-coupled receptors, NMDAR-induced signaling can also trigger eCB release, thereby suppressing the cannabinoid-sensitive synaptic transmission [173]. In summary, activated NMDA receptor induces the release of eCBs and CBR stimulation, which result in decreasing NMDA receptor activity and may prevent from excitotoxic insults. This regulatory relationship may have profound consequences not only for neuronal function, but also for many pathological events [171]. Under physiological conditions, eCBs can prevent from NMDA receptor overactivation and provide neuroprotection of neuronal cells [171,174]. The CB1R-dependent protection has been observed in dorsal root ganglion neurons, hippocampal slice cultures, murine cerebrocortical neurons cultured in vitro and mouse cerebral cortex in vivo [164,167,168,169,175,176,177,178].

## 4. Conclusions

Over the past few decades, a significant amount of work has been carried out to understand the regulatory function of endocannabinoids in CNS. The discovery of a role of eCBs within CNS as well as their potential therapeutic benefits has turned the attention into the use of cannabinoids for medical purposes. The cannabinoids are now classified as endocannabinoids (naturally produced in human body), phytocannabinoids (present in plants) and synthetic cannabinoids (produced chemically) [179]. Although over 60 different types of pharmacologically active cannabinoids have been isolated from the cannabis plant, their medical benefits are not fully elucidated. CB-based therapies have been studied for a variety of neurodegenerative diseases, e.g., stroke, epilepsy, Huntington’s disease, multiple sclerosis, amyotrophic lateral sclerosis, Alzheimer’s or Parkinson’s diseases, sometimes with conflicting results related to the effects of CBs on neurotransmission and cellular mechanisms of neuroprotection [5,180]. Moreover, increasing access to cannabis and/or cannabinoids can result in serious side effects such as addiction, respiratory illness and decline in cognitive processing [181]. As we summarized above, eCB-mediated regulation of calcium channels in neural cells can be influenced by differently transmitted signals, generated by more or less specific receptors, buy evidently controlled by calcium. Further investigation is essential for better understanding of eCBs effects, downstream signaling pathways, neurobiological mechanisms of ECS and the role of calcium channels to develop more safe and effective medical therapy.

## Data Availability

The study did not report any data.
